# Supplementary material for: Structure of the Enterobacter pan-genome is revealed using machine learning
Source: Microbiol Spectr. 2025 Dec 15;14(2):e01922-25. doi: 10.1128/spectrum.01922-25 (PMC12889083; doi:10.1128/spectrum.01922-25)
Supplement: Supplemental Figures — Fig. S1 to S12. [file spectrum.01922-25-s0001.docx]

Supplemental Figures
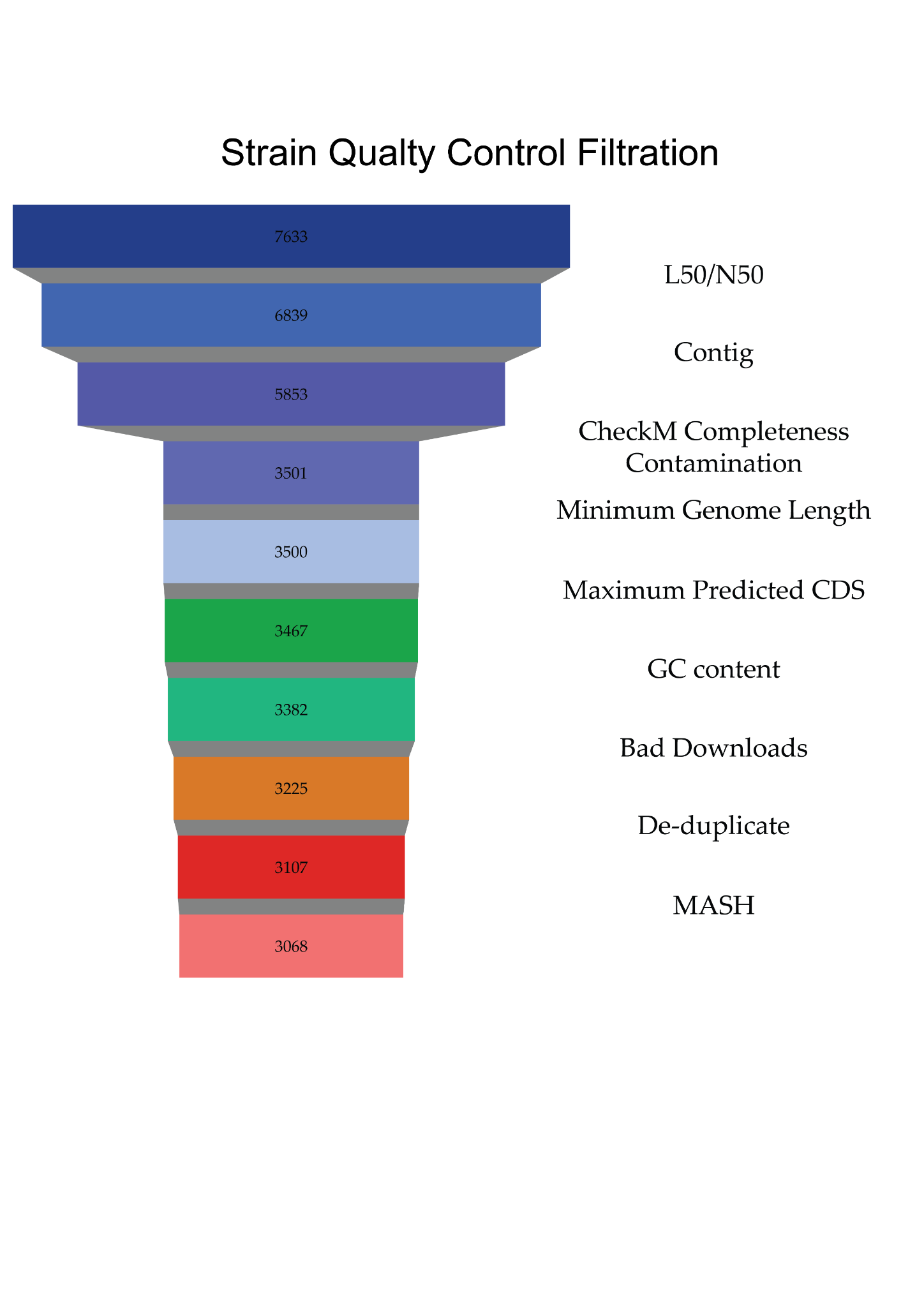


***Supplemental Figure 1 - Pipeline for the filtration of genome sequences:*** Filtration of genomes goes through the above steps. Most sequences are filtered out due to their checkM completeness score, too many contigs, or poor L50/N50 scores (see Methods). Of the 7,114 available sequences labeled as *Enterobacter* on BV-BRC, 2,785 passed all QC metrics and were used for downstream analysis. An additional 519 complete sequences were processed from NCBI Genomes Database and were subjected to filtration criteria, of which 283 were included in the final pangenome.

***Supplemental Figure 2 - Heaps’ Plot for the complete-genome pangenome:*** The core genome (purple) and accessory genome (orange) stabilize relatively quickly, with these two categories making up approximately 10^4^ of the genome in the pangenome. Most of the growth in
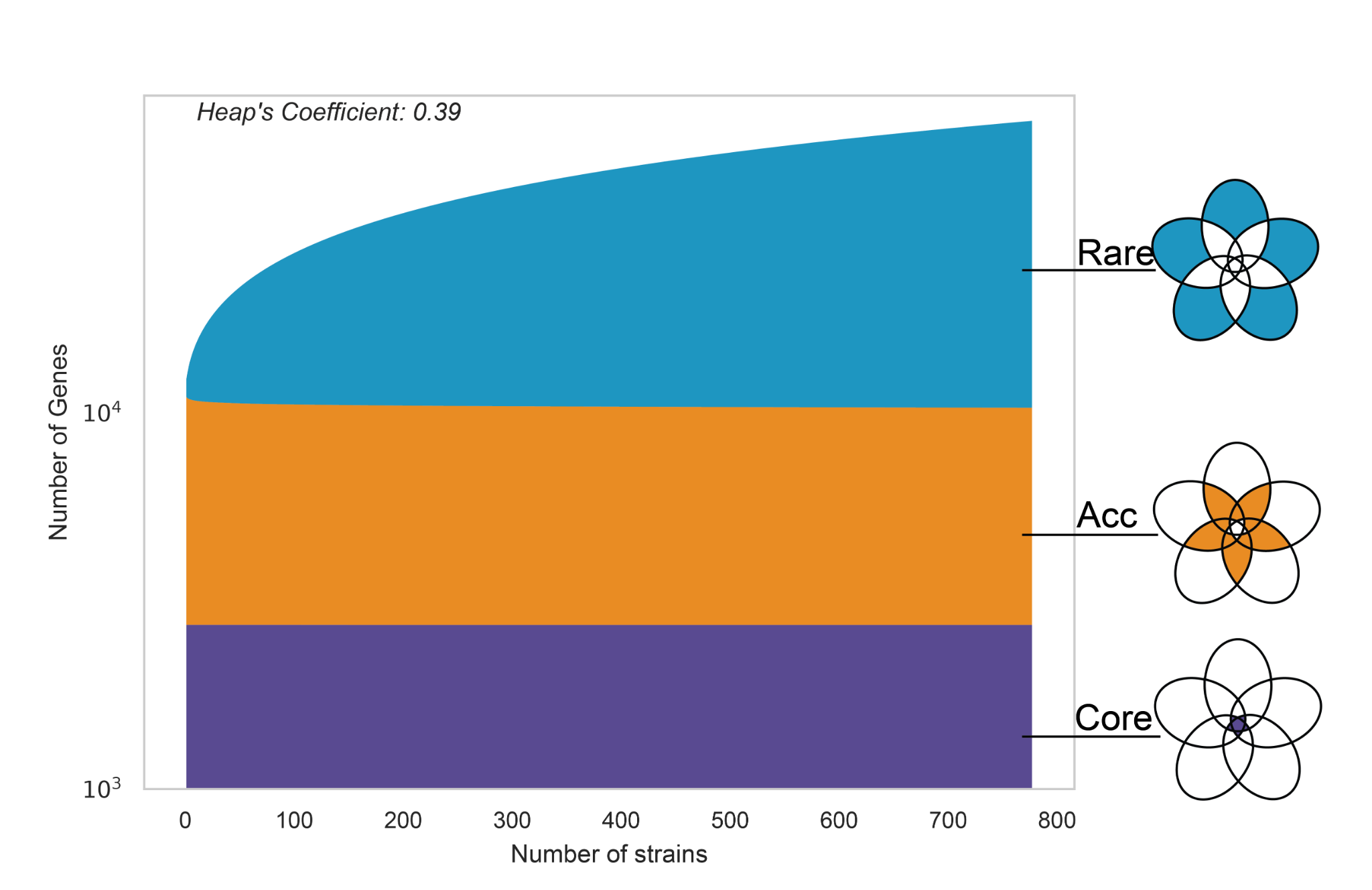
genes in the pangenome is in the rare genome, with each strain contributing an average of 65.75 new rare genes across the complete strains in the pangenome.

***Supplemental Figure 3 - Mash distance clustermap with annotated Mash clusters and most enriched type strain:*** The clustermap displays the pairwise Mash distance between all complete sequences in the pangenome, with the assigned cluster for each group of strains and the associated species for each Mash cluster. Clusters 1-12 are associated with *E. intestinhominis* (or *E. hormaechei subsp. hoffmannii*) and *E. hormaechei* subspecies while the remaining clusters are associated with other *Enterobacter* species.
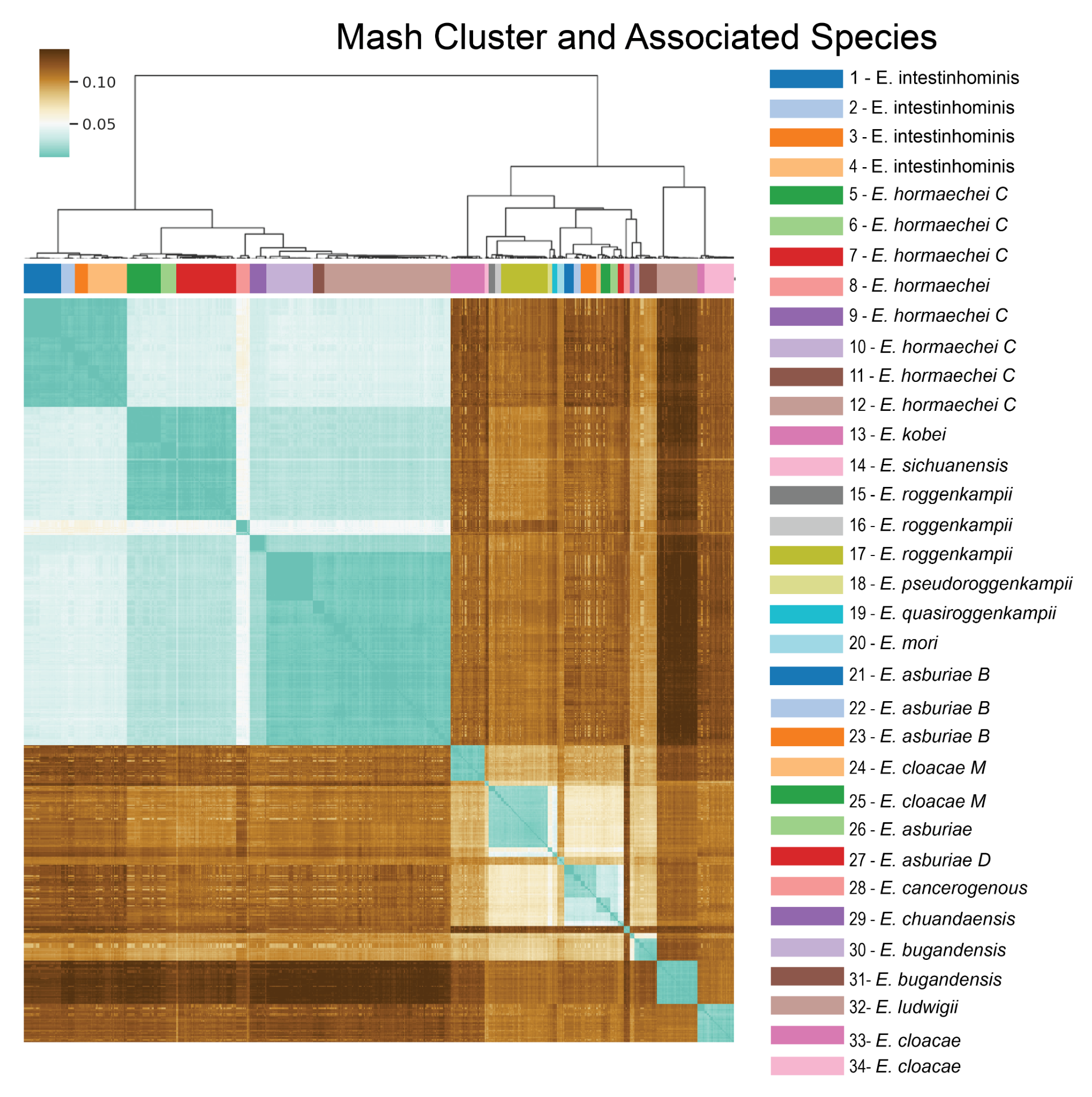


***Supplemental Figure 4 - Sunburst plots of the core, accessory, and rare genomes by COG categories:*** The core genome contains the fewest poorly characterized genes, while the accessory and rare genomes contain significantly more, with the rare genome being more than 68% composed of poorly characterized genes. The majority of genes in the core genome are associated with metabolic functions, while cellular processes and signaling make up a larger proportion of the accessory and rare genomes.
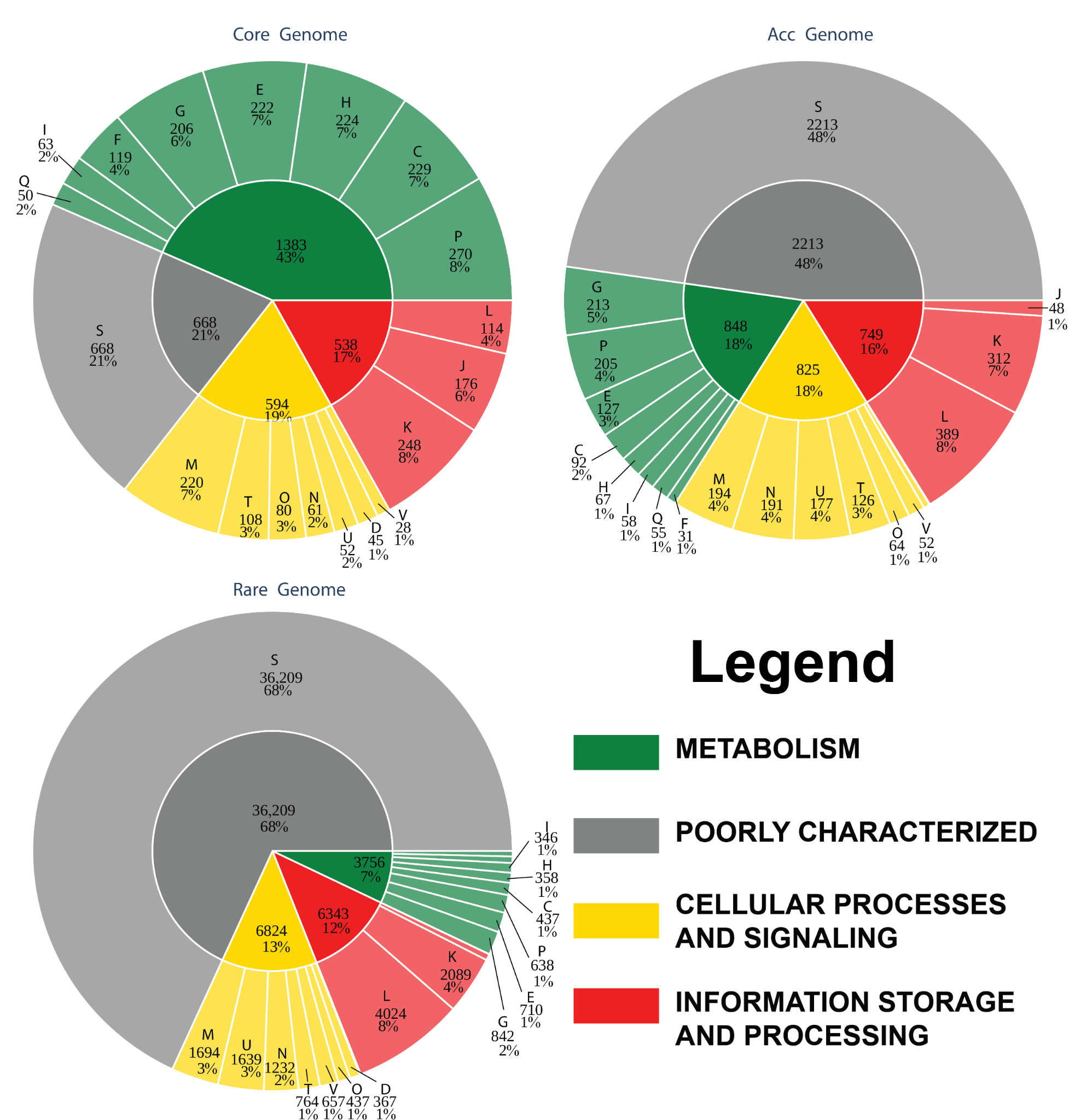


***Supplemental Figure 5 - Plot of allelic distribution of core genes and conservation of genes of different COG categories:* A)** Histogram of the number of alleles associated with a given core gene across the pangenome. Within the core genome, 472genes have an allele which is present in 50% or more of all genomes, with 45 of these genes having a single allele being found in 95% of all strains. **B)** Several COG categories display disproportionate conservation as compared to others in the core genome. Pairwise Z-tests for proportions were used to compare the content of the core, 50% dominant, and 95% dominant groups, with the larger groups having the smaller groups excluded for this comparison.
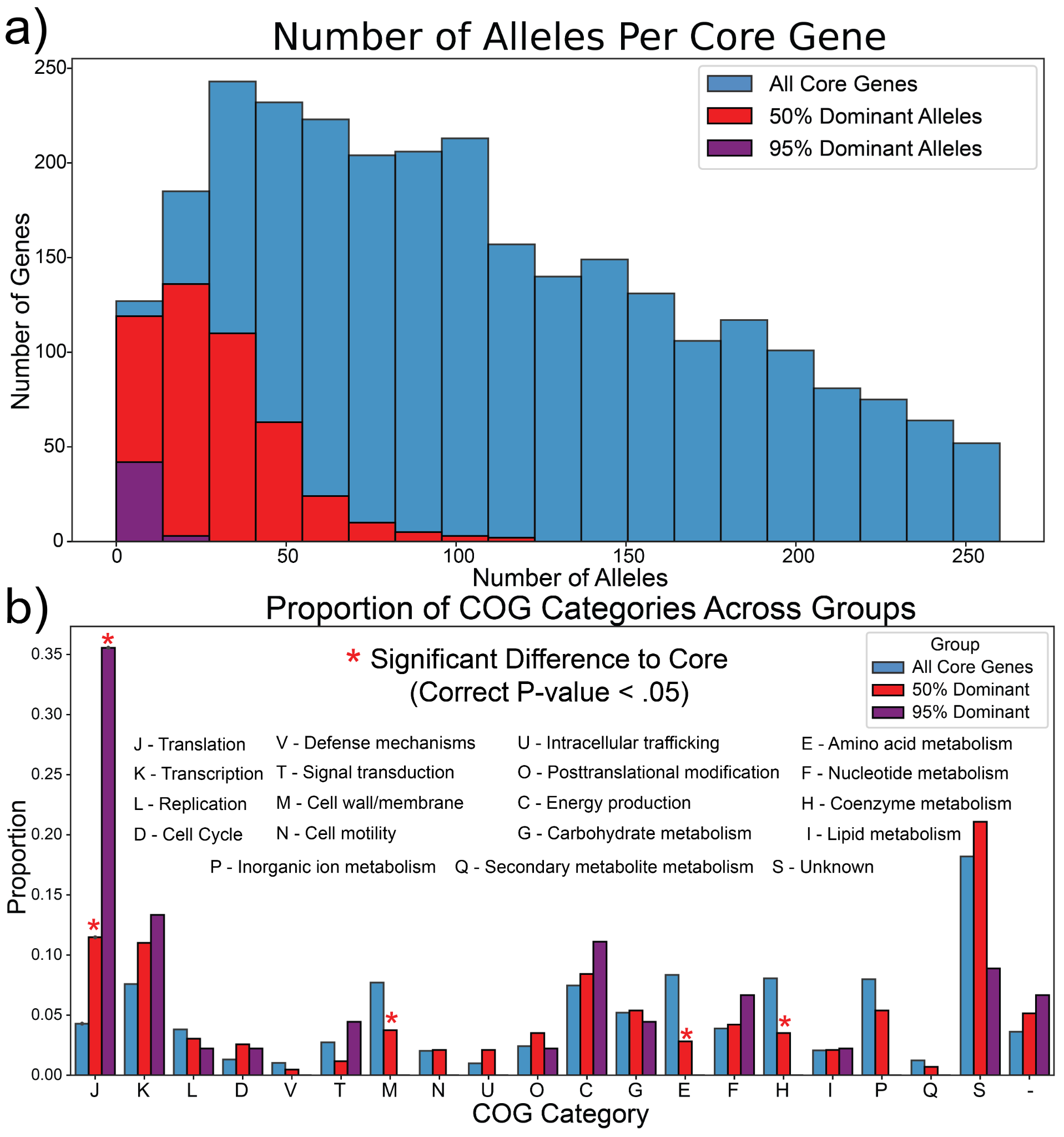


***
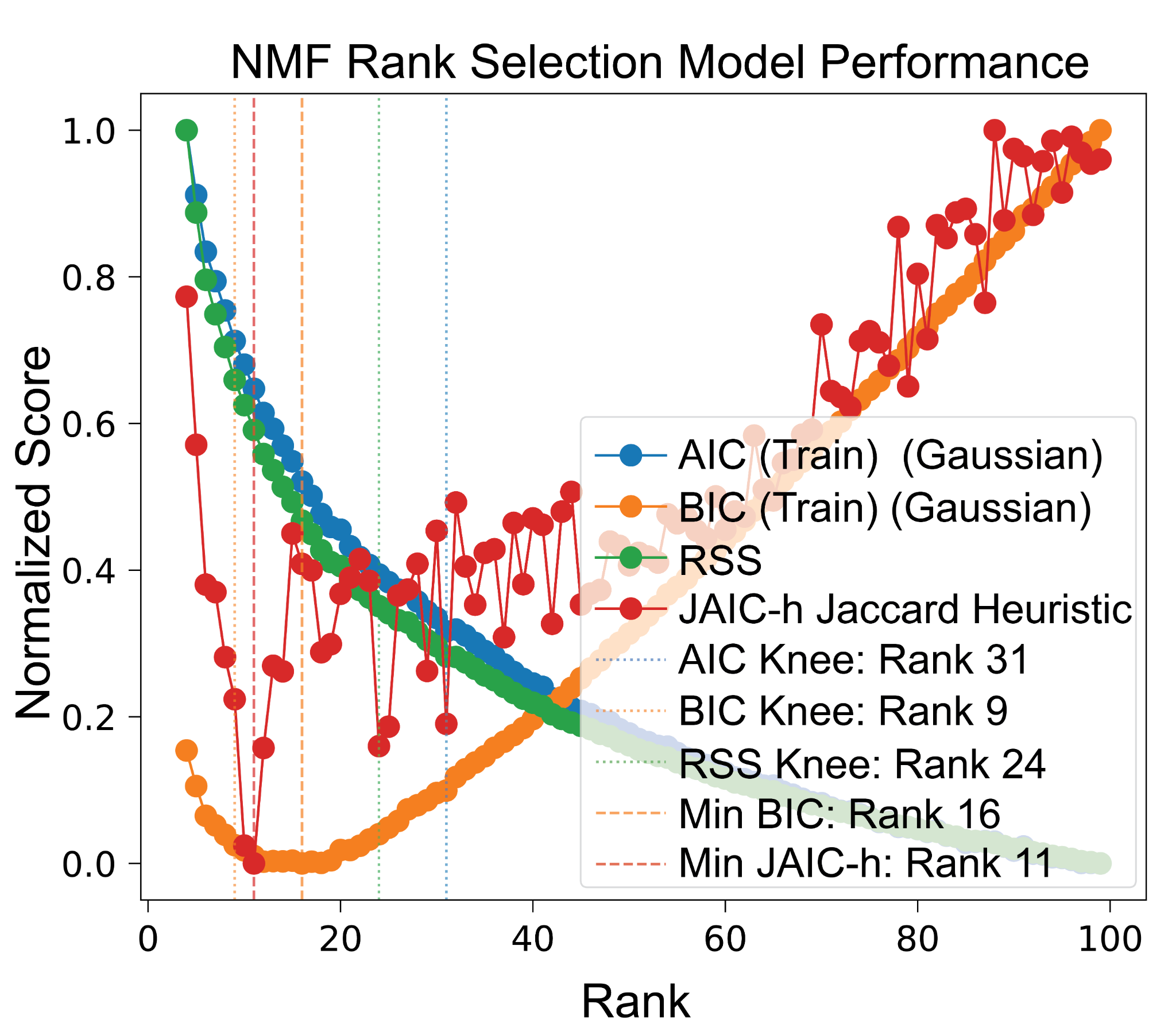
***

***Supplemental Figure 6 - Plot of reconstruction metrics across different dimensionalities of NMF:*** NMF was run on the dataset on ranks 0 to 99 , and performance metrics were calculated on each rank including the Akaike information criteria (AIC), Bayesian information criteria (BIC), residual sum of squares (RSS), and the Jaccard Heuristic (binarized) for AIC (JAIC-h). The minimum value represents the best performing value for a given metric, while the knee represents the point of diminishing returns for a given metric. The AIC knee was selected as the optimal rank of 31 in order to select a statistically robust model that has sufficiently high rank to represent the data while avoiding overdecomposition.

***
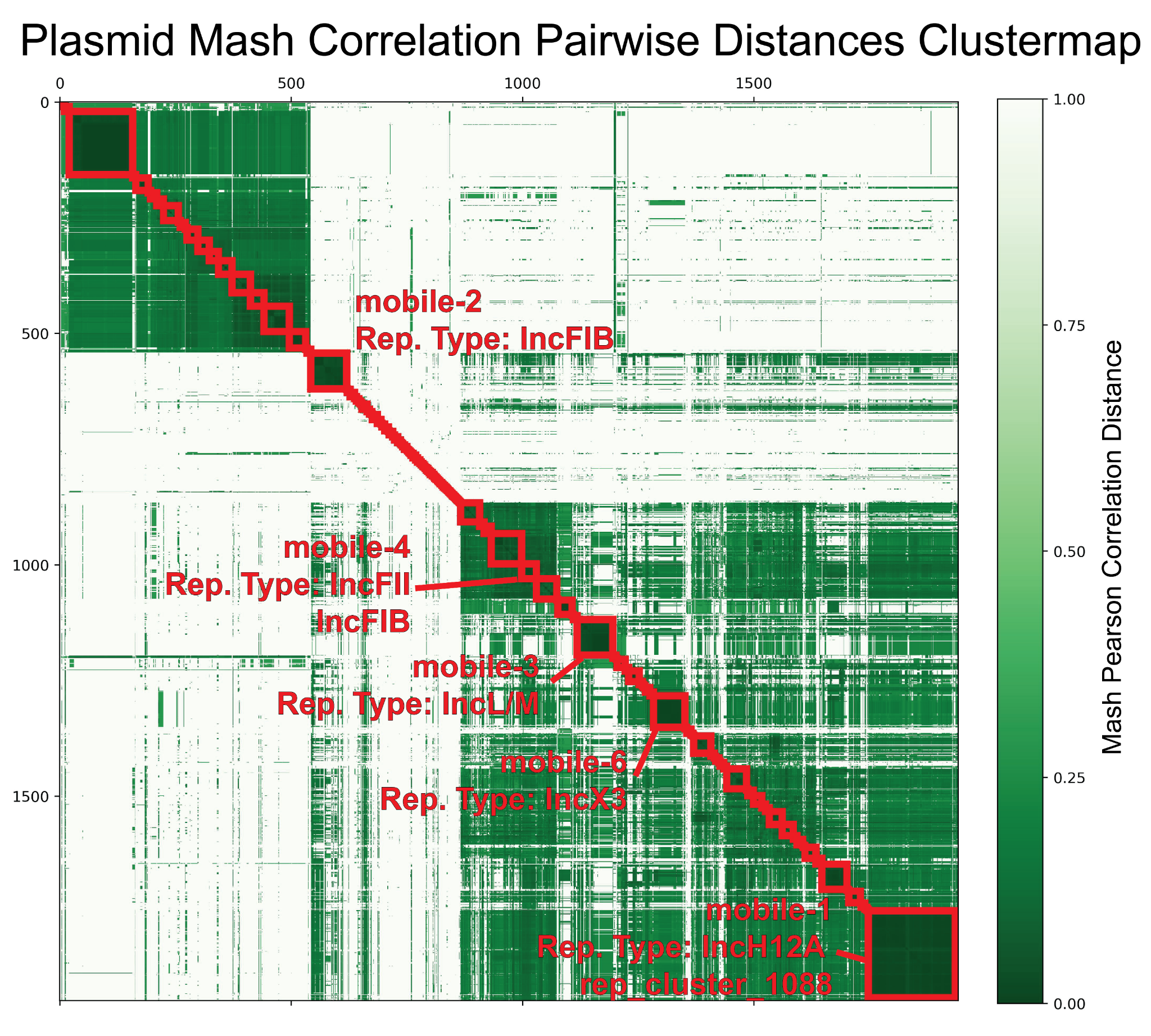
Supplemental Figure 7 - Mash Diagram of Plasmid Sequences:*** Pairwise clustering of all plasmid sequences. Clusters of sequences were calculated across the pangenome using the same clustering methods as for full strain sequence Mash analysis performed in this study. Five of the clusters had 90% or greater overlap with the strain membership of mobile phylons which are labeled on the plot adjacent to the corresponding cluster. Each of these clusters contained plasmids of which 90% or more had a specific replication type. Replication typing was performed using Mob Suite and the re_cluster_1088 is a plasmid cluster associated with the *E. coli* plasmid with GenBank accession KU353730.

***Supplemental Figure 8 - Sankey diagram displaying flow of strains from MASH clusters to associated non-mobile Phylons:*** Strains in each MASH cluster (left) are distributed across species-associated Phylons (right). All characterized Phylons have corresponding MASH clusters which share a species designation, while uncharacterized or mobile Phylons have associated strains from MASH clusters associated with multiple species. The “None” category for the Phylons represents strains which did not have sufficient threshold in a single Phylon to be classified as a member of that Phylon. This is representative of intermediate affinities for multiple Phylons, an example of which is a number of *hoffmannii* strains which show partial affinity for Phylons *hormaechei-stigerwaltii-1 and 3* but sufficient difference to these two major grouping as to not be a member of either.
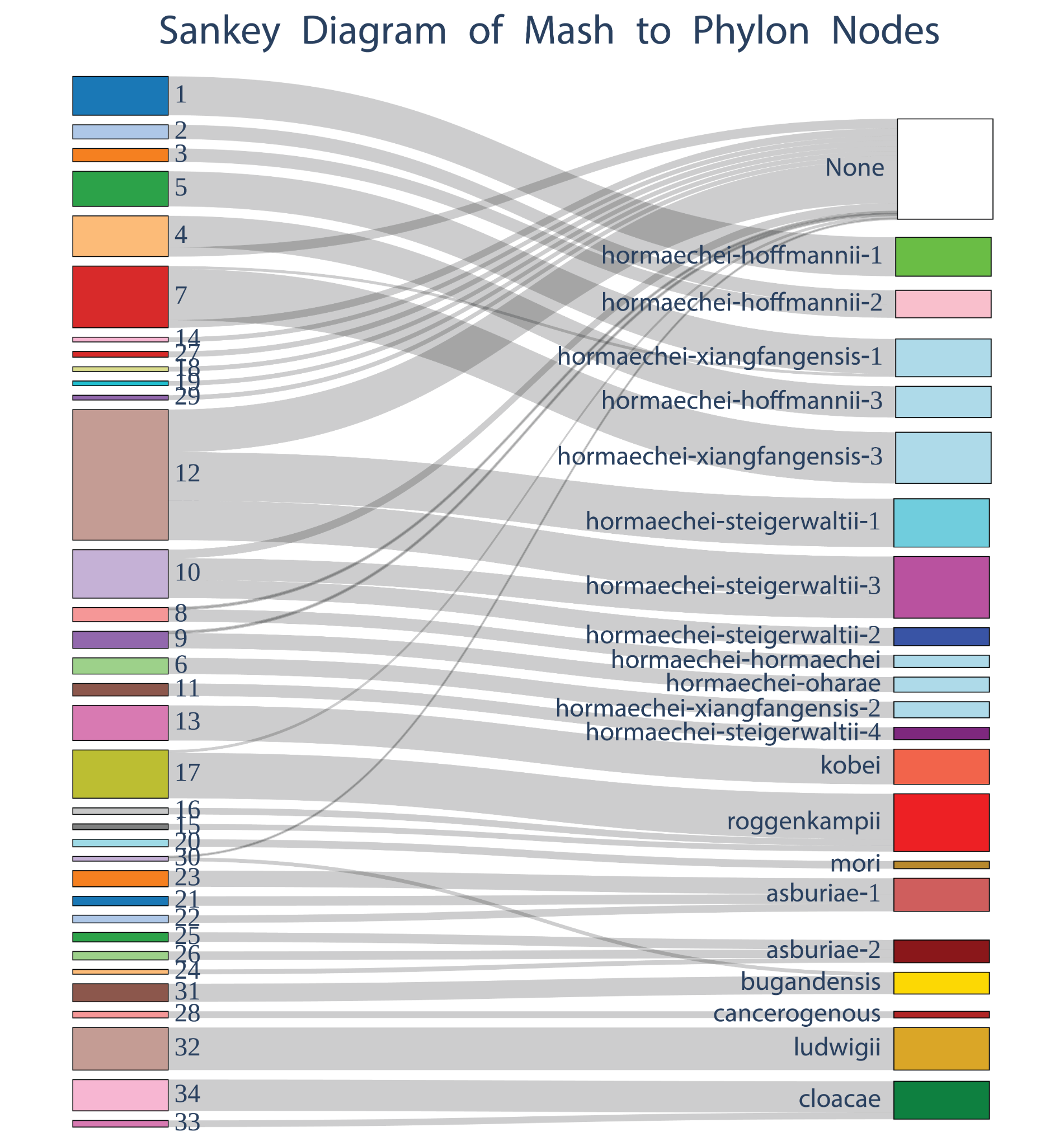


***Supplemental Figure 9 - Genome Diagram of Yersiniabactin Pathogenicity Islands:*** Enlarged panel from Fig. 5c. Strain neighborhoods are annotated with country of origin, date of isolation, and assigned Phylon. Red genes are virulence factors found in this pathogenicity island associated with Yersiniabactin production. Black and grey genes indicate other rare genes associated with this island with black genes being found with this island in all occurrences in the pangenome. Connections between genes on adjacent tracks indicates that the genes are homologous.
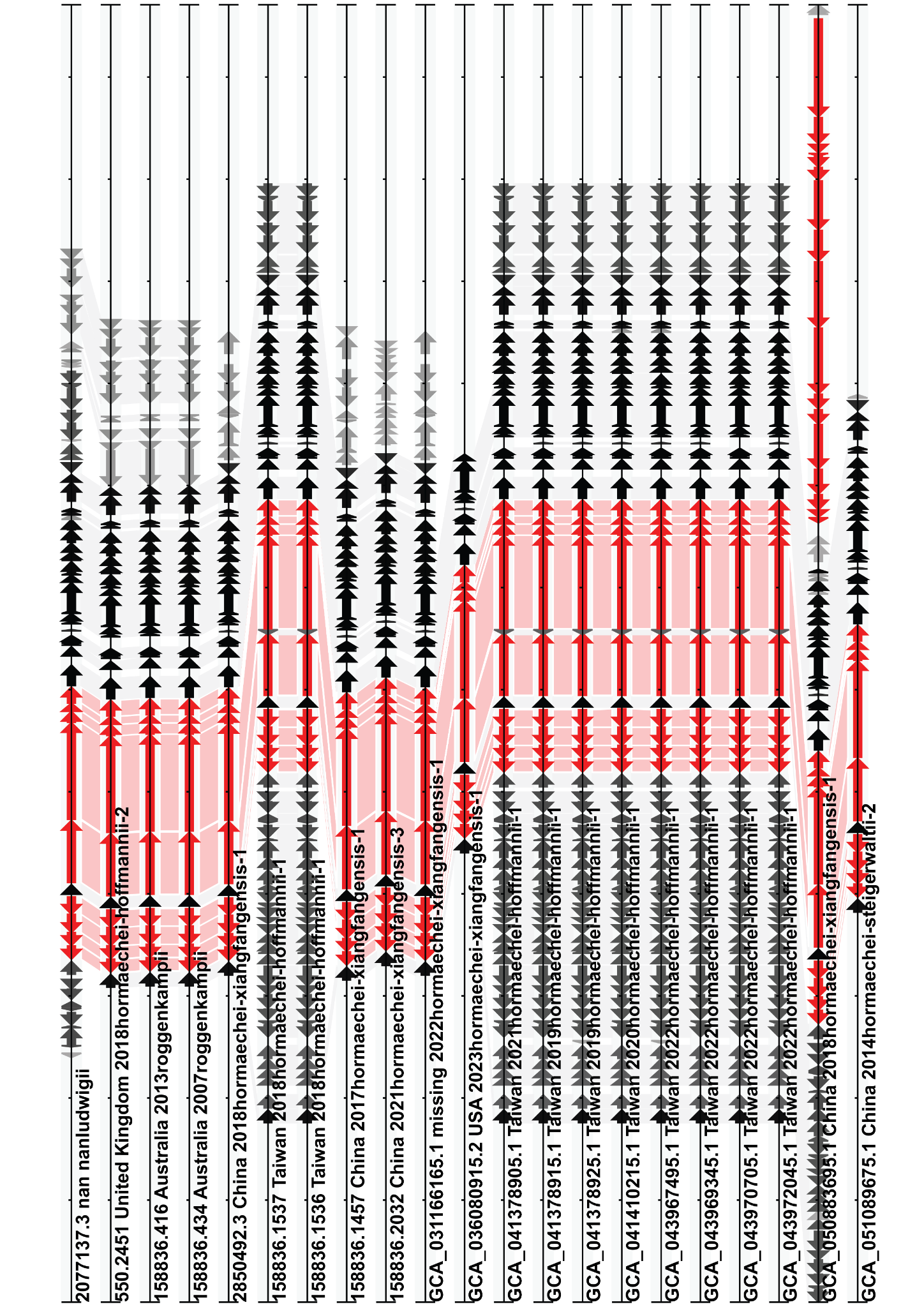


***Supplemental Figure 10 - Inferred affinities of 2,291 non-complete whole-genome sequences:*** Affinities for WGS strains to Phylons based on their genetic content were calculated (see Methods). Strains which showed a binarized affinity of one for a Phylon are summed to display in the above bar chart, with the mobile Phylons being colored by the corresponding characterized Phylon the strain is assigned to.
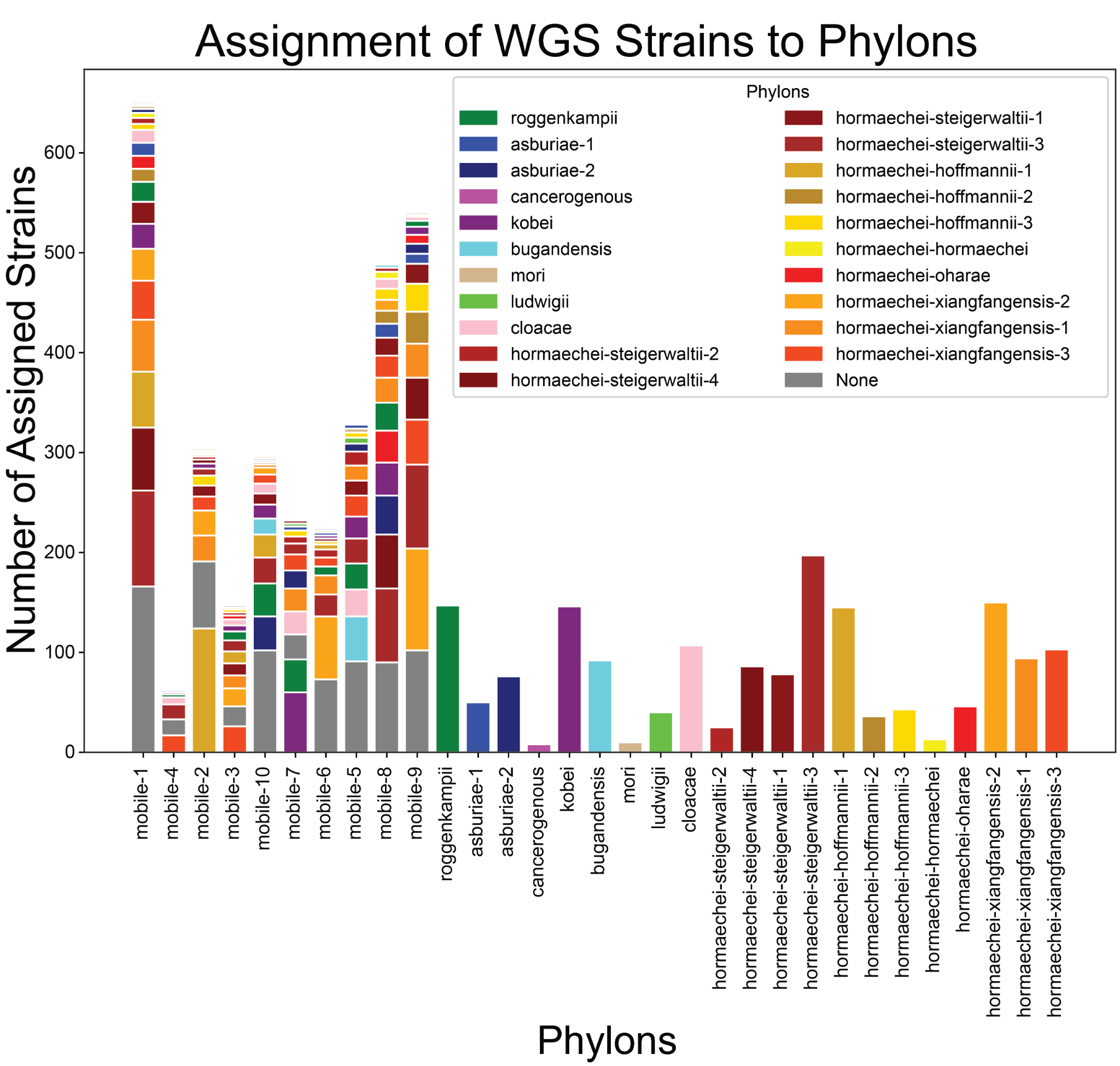


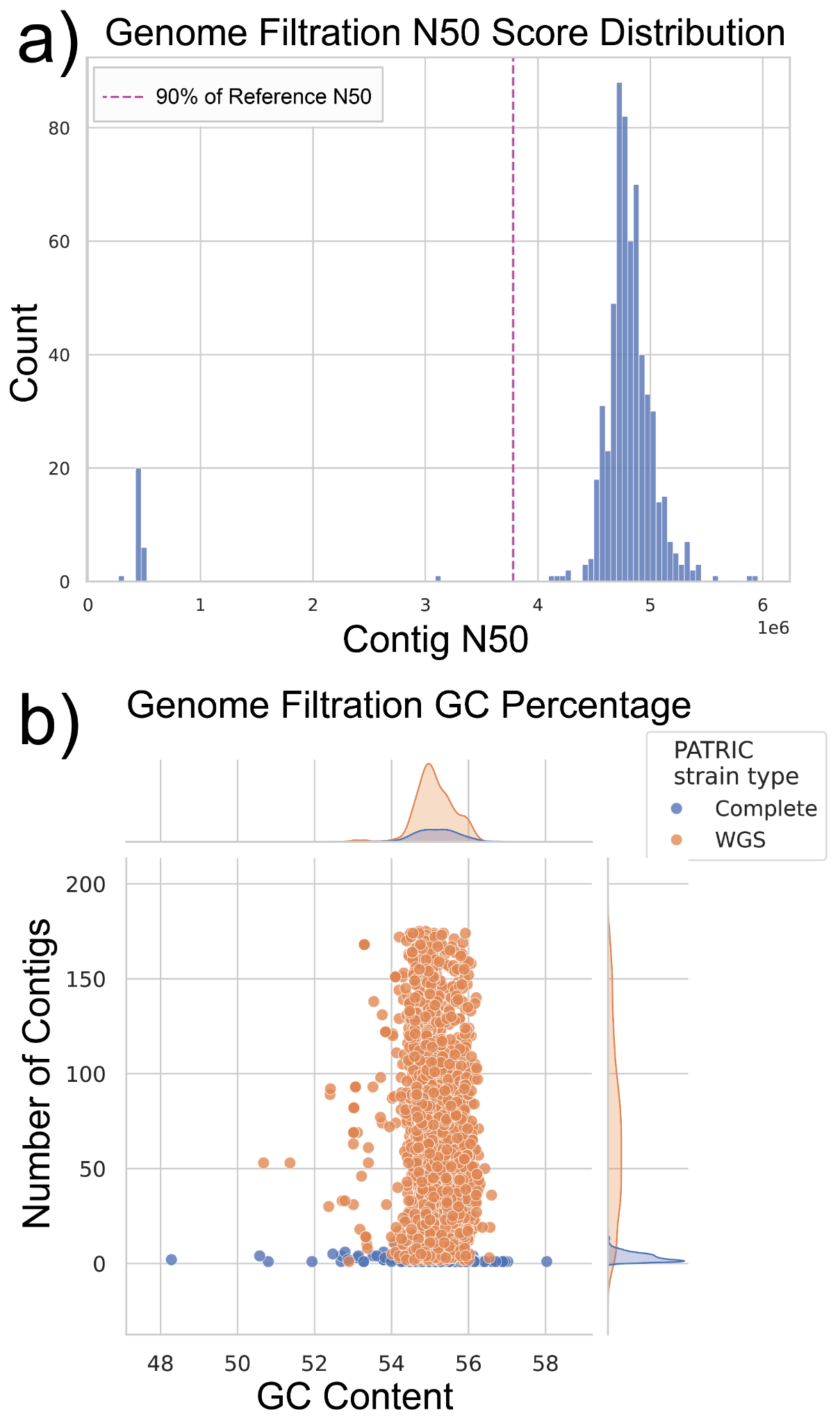


***Supplemental Figure 11 - GC Percentage and N50 Score Distributions for strains filtered for pangenomic analysis:* a)** Distribution of N50 Values of genomes filtered for use in the pangenome. All genomes with an N50 Score below 90% of the reference value were filtered out, a value of 3,780,000. The reference genome selected was *E. hormaechei* (assembly ASM1904824v1). **b)** The GC content distribution of genomes filtered for pangenome use. Genomes with less than 54% or greater than 57% GC content were excluded from inclusion in the pangenome.


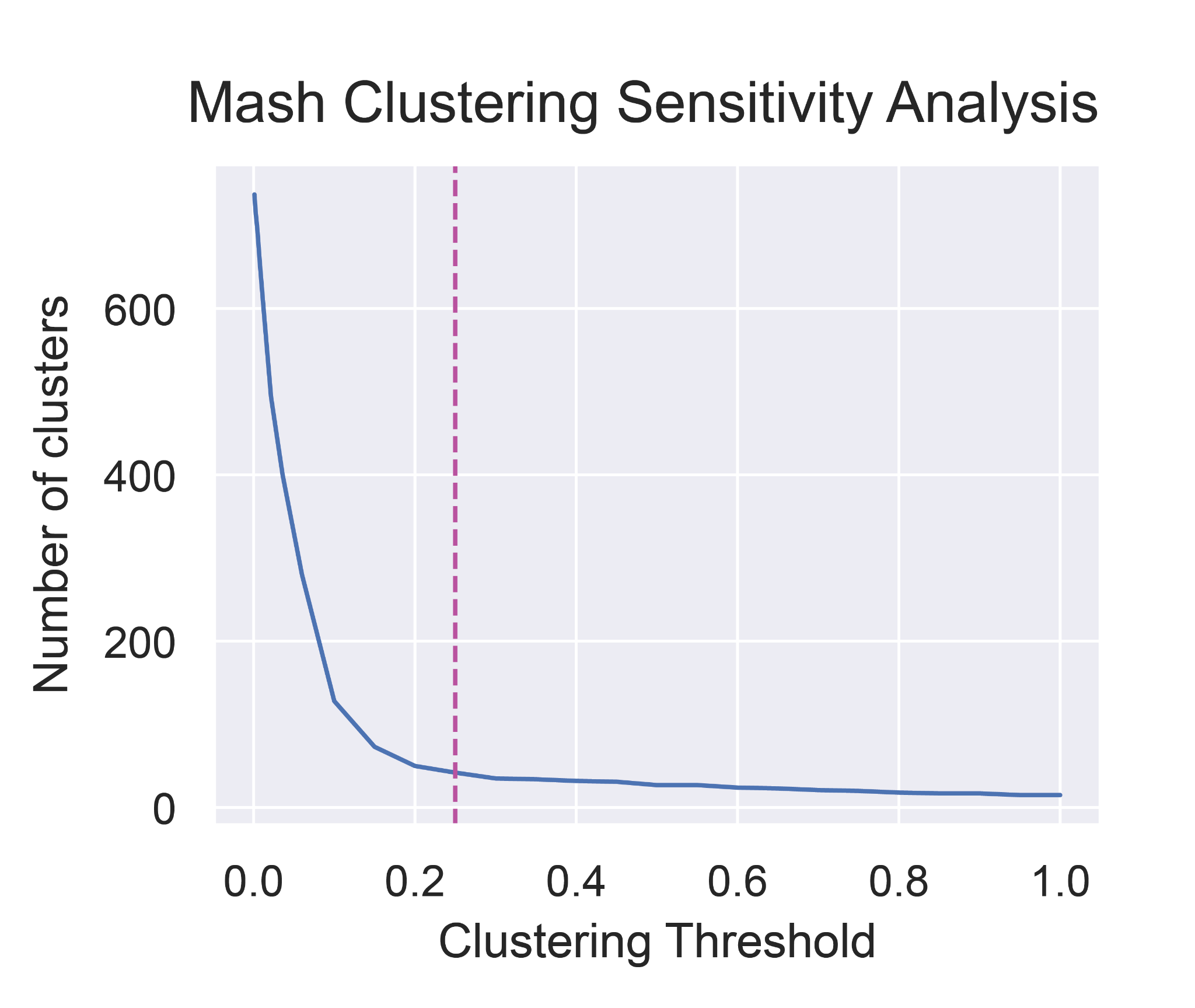


***Supplemental Figure 12 - Mash clustering threshold sensitivity analysis:*** Mash clustering was performed using Ward’s Minimum variance clustering on values of 1 - Pearson’s correlation coefficient for Mash distance between strains. The above plot displays a sensitivity analysis performed to determine the optimal clustering threshold. A value of .25 was selected as a conservative estimate of the elbow of the plot.
